# Work- and family-related stressors and risk of hazardous alcohol use: the role of social support. A cohort study in Sweden

**DOI:** 10.1093/alcalc/agaf058

**Published:** 2025-09-16

**Authors:** Ridwanul Amin, Julia Spaton Goppers, Jette Möller, Karin Engström, Anna Sidorchuk, Ellenor Mittendorfer-Rutz, Katalin Gémes

**Affiliations:** Department of Clinical Neuroscience, Division of Insurance Medicine, Karolinska Institutet, Berzelius väg 3, SE-171 77 Stockholm, Sweden; Department of Medicine, Division of Infectious Diseases, Karolinska Institutet, Akademiska Stråket 1, Bioclinicum J7:20, SE-171 64 Stockholm, Sweden; Department of Clinical Neuroscience, Division of Insurance Medicine, Karolinska Institutet, Berzelius väg 3, SE-171 77 Stockholm, Sweden; Department of Global Public Health, Karolinska Institutet, Solnavägen 1E, SE-17 177, Stockholm, Sweden; Department of Global Public Health, Karolinska Institutet, Solnavägen 1E, SE-17 177, Stockholm, Sweden; Department of Clinical Neuroscience, Centre for Psychiatry Research, Karolinska Institutet, Norra Stationsgatan 69, SE-113 64 Stockholm, Sweden; Stockholm Health Care Services, Region Stockholm, Solnavägen 1E, SE-17 177 Stockholm, Sweden; Department of Clinical Neuroscience, Division of Insurance Medicine, Karolinska Institutet, Berzelius väg 3, SE-171 77 Stockholm, Sweden; Department of Clinical Neuroscience, Division of Insurance Medicine, Karolinska Institutet, Berzelius väg 3, SE-171 77 Stockholm, Sweden

**Keywords:** stressful event, job strain, AUDIT, alcohol use disorder, cohort, population survey

## Abstract

**Aims:**

To investigate whether work- and family-related stressors increase the risk of hazardous alcohol use among low-risk drinkers, and to examine the role of sex and social support.

**Methods:**

Overall, 4046 individuals aged 25–55 years, living and working in Stockholm, without a history of hazardous alcohol use, who completed the Mental Health, Work and Social Relations study questionnaire on work- and family-related stressors (exposures) during 1998–2000 and 2001–03, were included. This cohort was followed until 2010 for hazardous alcohol use (outcome) assessed by the Alcohol Use Disorder Identification Test. Weights for selective attrition were calculated, and crude and multivariate (adjusting for sociodemographic, health-, and baseline stress-related factors) logistic regression models, yielding odds ratios (ORs) with 95% confidence intervals (CIs), were used to estimate the exposure–outcome associations. The analyses were stratified by sex and social support.

**Results:**

The crude OR for hazardous alcohol use was 1.28 (95% CI: .88–1.88) and 1.48 (95% CI: 1.05–2.08) among individuals with work-related and family-related stressors, respectively, when compared with those without these exposures. The association between family-related stressors and hazardous alcohol use was slightly more pronounced among women (crude OR, 95% CI: 1.66, 1.02–2.71) and those with low social support (crude OR, 95% CI: 2.06, 1.17–3.62). Adjusting for the history of previous work- and family-related stressors explained most of the associations.

**Conclusions:**

In this population-based longitudinal study of individuals without a history of hazardous alcohol use, we found greater vulnerability to transitioning into hazardous alcohol use among those who experienced family-related stressors, particularly women and individuals with low social support.

## Teaser

**Short summary:** Our population-based longitudinal study of individuals without a history of hazardous alcohol use found that experiencing family-related stressors was associated with a higher risk of hazardous alcohol use during the follow-up. This association was slightly more pronounced among women and those with low social support.

## Introduction

Extensive alcohol consumption is associated with several adverse health and societal consequences (Agardh *et al*. 2016). On average, the Organisation for Economic Co-operation and Development (OECD) countries, including Sweden, spend 2.4% of their health expenditure on addressing harms inflicted by alcohol consumption, and the proportion of the population who are alcohol dependent ranges from 3.7% to 10% (Organisation for Economic Co-Operation and Development, 2021). However, in many Western countries, habitual alcohol consumption is culturally embedded in many social contexts (Reducing Alcohol Related Harm (RARHA), 2016). Overall, 75%–80% of the population without alcohol use disorder (AUD) has reported some alcohol drinking in Sweden (Centralförbundet för Alkohol-Och Narkotikaupplysning (CAN), 2024). While there are no safe levels of alcohol use, guidelines often refer to consumption limits and patterns that are associated with a low risk of adverse health outcomes (World Health Organization, 2023). Individuals who exceed the recommended consumption limits, given their higher risk of adverse health outcomes, are often referred to as persons with hazardous alcohol use. The current Swedish recommendations define hazardous alcohol use as an intake of more than ten standard drinks per week (one standard drink equals 12 g of 100% alcohol) or heavy episodic drinking occasions (i.e. binge drinking of more than four drinks per occasion) (National Board of Health and Welfare, 2023). One of the major public health concerns regarding habitual alcohol consumption is that low-risk drinkers may be at risk of increasing their alcohol use and developing hazardous alcohol use habits (World Health Organization, 2023, National Board of Health and Welfare, 2023).

One of the reasons why low-risk drinkers may enter hazardous alcohol use is a maladaptive response to psychological distress (Dawson *et al*. 2005). Psychological distress, often referred to as negative stress or simply stress, occurs when the stressor triggers a negative emotional response or emotional state. According to the allostatic load theory, a chronic stressor requires constant and repeated adaptation of the individuals, which, in the long term, can exceed the coping ability of the individual and lead to disease or the use of maladaptive coping strategies such as alcohol use (Mcewen and Stellar 1993, D’Aquino and Callinan 2024). A combination of alcohol’s neurobiological effects—such as increased stress sensitivity—and its use as a maladaptive coping strategy in response to allostatic load can contribute to the development of hazardous drinking (Becker 2017, Wittgens *et al*. 2022). Work- and family-related stressors are common sources of allostatic load, and their associations with problem drinking have been reported in previous research (Dawson *et al*. 2005, Keyes *et al*. 2012, Pilling *et al*. 2012, Almroth *et al*. 2022, Pan *et al*. 2023).

One of the most widely used work-related stress models is the “Job-Demand-Control-Support model,” developed by Karasek and Theorell in 1990 (Karasek RA, 1990). According to the model, high demands at work, such as heavy workload together with poor control over the work tasks, are considered as the most stressful work characteristics and referred to as high “job strain.” In previous longitudinal studies in the field, high job strain was associated with higher alcohol-related morbidity among men and mortality among women (Almroth *et al*. 2022, Pan *et al*. 2023). Several family-related stressors have been identified in relation to harmful alcohol use in previous research. Going through divorce, marital dissatisfaction, death and illness of a close relative, and being part of violent conflicts, criminal victimization of self or a family member, and major financial crises were associated with alcohol-related problems, such as heavy drinking (i.e. >14 drinks/week for women, >21 drinks/week for men) and AUD in longitudinal studies (Dawson *et al*. 2005, Keyes *et al*. 2011, Pilling *et al*. 2012).

Most previous studies investigating the role of work- and family-related stressors in the development of hazardous alcohol use were cross-sectional, focused on the association between one specific work- or family-related stressor, examined only heavy drinking or AUDs, and lacked information on prior AUDs. However, from a public health perspective, it is important to understand the role of work- and family-related stressors in the transition from low-risk drinking to hazardous alcohol use, in order to identify less severe alcohol-related problems for early intervention and to prevent the development of AUDs.

Therefore, we aim to investigate how work- and family-related stressors contribute to the development of hazardous alcohol use among individuals without a prior history of hazardous alcohol consumption. Furthermore, to test the allostatic theory, we will not only consider the type of stressor but also adjust for the history of stressors, which makes it possible to distinguish the effect of recent stressors from more chronic ones. Based on the allostatic load theory, we hypothesize that adjustment for the history of the stressors will explain a substantial part of their effect on hazardous alcohol use.

### Sex differences

Previous research highlighted the neurobiological and social differences in relation to the psychological distress and alcohol use between women and men. Previous research found that women are more likely to initiate drinking to relieve psychological distress and are more sensitive to alcohol-induced neurodegeneration (Peltier *et al*. 2019). Consequently, higher allostatic load due to stress and alcohol use may lead to a more pronounced risk of developing hazardous alcohol use in response to work- and family-related stress among women compared to men (Peltier *et al*. 2019). Furthermore, among problem drinkers, men more often report financial and friendship-related stressors while women report stressors related to spouse and family relationships, which might reflect the different prevalence of family and work-related stressors in the lives of women and men (Brennan *et al*. 1993).

The extent to which work- and family-related factors contribute to developing hazardous alcohol use in women and men without prior hazardous alcohol use is not known, but we hypothesize that women are more likely to develop hazardous alcohol use due to work and especially family-related stressors than men, given the previously described higher stress sensitivity.

### Role of social support

Perceived social support may also influence how individuals change their alcohol use behavior as a response to psychological distress (Brooks *et al*. 2017). Social support can be defined as “the social resources that a person perceives to be available or that are actually provided to them by nonprofessionals in the context of both formal support groups and informal helping relationships” (Cobb 1976). It is commonly measured both quantitatively (instrumental support)—such as the number of regular social contacts or the availability of financial or physical assistance—and qualitatively (emotional support), which refers to the perceived quality of support, feelings of being cared for, and a sense of belonging to a social network (Cobb 1976, Moon *et al*. 2019). The presence of social support has been shown to help individuals with AUD cope with perceived stress (Humphreys *et al*. 1999), adhere to recovery plans (Moon *et al*. 2019), and find motivation to reduce harmful drinking habits (Moon *et al*. 2019). Both quantitative and especially qualitative social support have been shown to help substance use recovery (Weston *et al*. 2018). However, given the social aspects of drinking, the role of quantitative and qualitative social support in the association may differ. Therefore, we hypothesize that social support might mitigate the association between work- and family-related stress and hazardous alcohol use and, for those with higher social support, especially qualitative social support, the association between work- and family-related stress will be weaker than among those with low social support. Therefore, we aimed to advance the field by examining the longitudinal association between work- and family-related stressors and risk of hazardous alcohol use over time among individuals with no prior history of hazardous drinking and by exploring the moderating roles of sex and social support in these associations.

## Materials and Methods

### Study population, sample, and study design

The study sample was drawn from the PART study (Swedish acronym for Mental Health, Work and Social Relations), a population-based survey conducted between 1998 and 2000 (PART1) among Stockholm County residents aged 20–64 years with follow-ups in 2001–03, 2010, and 2021 (PART2–PART4, respectively). From the 19 742 randomly selected individuals who were invited to PART1, 10 441 individuals participated. Among those who participated in PART1, 8631 and 5621 individuals later participated in PART2 and PART3, respectively. Factors associated with nonparticipation and attrition are described in more detail in the supplementary methods.

**Figure 1:**
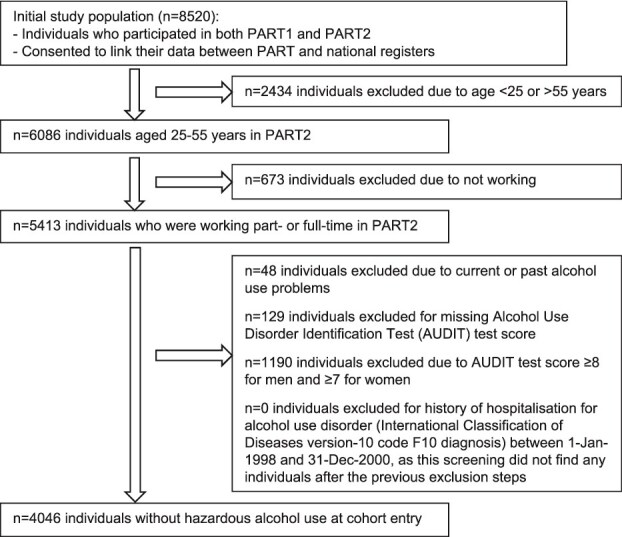
Flowchart of the selection of the study population.

In this study, we included individuals who gave informed consent to participate in both PART1 and PART2 and consented to data linkage of national registers (*n* = 8520), aged 25–55 years at PART1 (*n* = 6086), and who were working full- or part-time in PART2 (*n* = 5413). After excluding individuals with hazardous alcohol use at baseline (Fig. 1), the final sample included 4046 individuals.

### Exposures (PART2)

Work-related stress was assessed by the Karasek-Theorell’s job-demand-control survey, and dichotomized as the presence or absence of job strain, with job strain characterized by high demands and low control (Karasek, 1990). Family-related stress was dichotomized as either present or absent and was based on the stressful life event survey (Theorell *et al*. 1975). Details on the dichotomization and questionnaires are provided in supplementary methods and Supplementary Table S1.

### Outcomes

Hazardous alcohol use was defined as an AUDIT score of ≥8 in men and a score of ≥7 in women in PART3 (Lundin *et al*. 2015). This cut-off was validated in the Swedish general population as a screening tool for hazardous alcohol use, and it has been used in the Swedish health care system as a screening tool and as eligibility criteria for interventions to prevent AUDs (Reinert and Allen 2007, Lundin *et al*. 2015).

### Covariates and effect modifiers

Sociodemographic, health-, and previous stress-related covariates and their categorization are presented in Supplementary Table S1. Sex and social support were considered as effect modifiers. Social support was measured using Availability of Social Integration (AVSI) and Availability of Social Attachment (AVAT) scales and was dichotomized (high, low AVAT and high, low AVSI) at median.

### Statistical analysis

Missing values for covariates and effect modifiers were imputed, and individuals with missing data on work-related stress at PART2 (*n* = 95) and individuals with missing data on AUDIT in PART3 (*n* = 1723) were excluded from the respective analyses. We calculated Inverse Probability of Censoring Weights (IPCW) to handle potential selective attrition between PART2 and PART3.

To estimate the association between work- and family-related stress and hazardous alcohol use, we constructed IPCW weighted logistic regression models (one unadjusted/crude and three multivariate-adjusted), yielding odds ratios (OR) and adjusted odds ratios (AOR), respectively, with corresponding 95% confidence intervals (CIs). Multivariate-adjusted models were adjusted stepwise: sociodemographic variables (Model 1); health- and previous stress-related variables (Model 2); and social support (Model 3) (please see Supplementary Table S5). Effect modification by sex and social support was investigated by stratified analysis and by adding an interaction term to the models. We used SAS 9.4 for all analyses.

### Secondary analyses

First, to minimize selective attrition, we defined the outcomes as diagnosis of AUD (ICD-10 code F10) between 1 January 2004 and 31 December 2014 in inpatient or specialized outpatient records (Supplement method). Second, to investigate if stress from both exposures increases the risk of hazardous alcohol use, the risk of hazardous alcohol use was estimated among individuals exposed to only job strain, to only family-related stress, and to both job strain and family-related stress, compared to no job strain or family-related stress.

## Results

### Main analysis

The baseline characteristics of the study participants are presented in Table 1. Altogether, 2323 (57%) individuals had valid AUDIT data in 2010, of which 155 individuals were identified as hazardous drinkers.

**Table 1:** Descriptive characteristics of the study population (*n* = 4046) by job strain and any family-related stress

	All, *n* (%)	Work-related stress (job strain^a^), *n* (%)			Family-related stress^b^, *n* (%)	
Characteristics	4046 (100)	No 3092 (76.4)	Yes 859 (21.2)	Missing 95 (2.3)	No 1743 (43.1)	Yes 2303 (56.9)
*Sociodemographic factors*						
Sex						
Men	1568 (38.8)	1265 (40.9)	277 (32.2)	26 (27.4)	741 (42.5)	827 (35.9)
Women	2478 (61.2)	1827 (59.1)	582 (67.8)	69 (72.6)	1002 (57.5)	1476 (64.1)
Age (years)						
25–29	477 (11.8)	367 (11.9)	102 (11.9)	8 (8.4)	189 (10.8)	288 (12.5)
30–34	684 (16.9)	491 (15.9)	174 (20.3)	19 (2.0)	297 (17.0)	387 (16.8)
35–39	723 (17.9)	568 (18.4)	136 (15.8)	19 (2.0)	317 (18.2)	406 (17.6)
40–44	626 (15.5)	477 (15.4)	136 (15.8)	13 (13.7)	259 (14.9)	367 (15.9)
45–49	669 (16.5)	519 (16.8)	134 (15.6)	16 (16.8)	284 (16.3)	385 (16.7)
50–55	867 (21.4)	670 (21.7)	177 (20.6)	20 (21.1)	397 (22.8)	470 (20.4)
Educational level (years)						
Compulsory school (0–9)	371 (9.2)	255 (8.2)	100 (11.6)	16 (16.8)	148 (8.5)	223 (9.7)
Upper secondary school (10–12)	1711 (42.3)	1288 (41.7)	386 (44.9)	37 (38.9)	743 (42.6)	968 (42.0)
College/university (>12)	1960 (48.4)	1546 (50.0)	372 (43.3)	42 (44.2)	851 (48.8)	1109 (48.2)
Missing	4 (.1)	3 (.1)	1 (.1)		1 (.1)	3 (.1)
Country of birth						
Sweden	3700 (91.4)	2860 (92.5)	760 (88.5)	80 (84.2)	1610 (92.4)	2090 (90.8)
Other countries	343 (8.5)	229 (7.4)	99 (11.5)	15 (15.8)	132 (7.6)	211 (9.2)
Missing	3 (.1)	3 (.1)			1 (.1)	2 (.1)
Living with another adult						
No	974 (24.1)	715 (23.1)	230 (26.8)	29 (30.5)	388 (22.3)	586 (25.4)
Yes	3071 (75.9)	2376 (76.8)	629 (73.2)	66 (69.5)	1355 (77.7)	1716 (74.5)
Missing	1 (<.1)	1 (<.1)				1 (<.1)
Living with a child						
No	1518 (37.5)	1150 (37.2)	340 (39.6)	28 (29.5)	688 (39.5)	830 (36.0)
Yes	2523 (62.4)	1938 (62.7)	519 (60.4)	66 (69.5)	1053 (60.4)	1470 (63.8)
Missing	5 (.1)	4 (.1)		1 (1.1)	2 (.1)	3 (.1)
Financial difficulties in last 12 months						
No	3391 (83.8)	2651 (85.7)	671 (78.1)	69 (72.6)	1522 (87.3)	1869 (81.2)
Yes	655 (16.2)	441 (14.3)	188 (21.9)	26 (27.4)	221 (12.7)	434 (18.8)
*Health- and prior stress-related factors*						
Self-reported health						
Average to very good	3786 (93.6)	2931 (94.8)	771 (89.8)	84 (88.4)	1662 (95.4)	2124 (92.2)
Bad or very bad	251 (6.2)	155 (5.0)	85 (9.9)	11 (11.6)	77 (4.4)	174 (7.6)
Missing	9 (.2)	6 (.2)	3 (.3)		4 (.2)	5 (.2)
Previous healthcare use for mental disorders^c^						
No	4037 (99.8)	3086 (99.8)	857 (99.8)	94 (98.9)	1742 (99.9)	2295 (99.7)
Yes	9 (.2)	6 (.2)	2 (.2)	1 (1.1)	1 (.1)	8 (.3)
Previous healthcare use for somatic disorders^c^						
No	3696 (91.3)	2832 (91.6)	782 (91.0)	82 (86.3)	1610 (92.4)	2086 (90.6)
Yes	350 (8.7)	260 (8.4)	77 (9.0)	13 (13.7)	133 (7.6)	217 (9.4)
Previous job strain						
No	2988 (73.9)	2626 (84.9)	341 (39.7)	21 (22.1)	1316 (75.5)	1672 (72.6)
Yes	672 (16.6)	222 (7.2)	447 (52.0)	3 (3.2)	279 (16.0)	393 (17.1)
Missing	386 (9.5)	244 (7.9)	71 (8.3)	71 (74.7)	148 (8.5)	238 (10.3)
Previous family-related stress						
No	1629 (40.3)	1272 (41.1)	324 (37.7)	33 (34.7)	883 (50.7)	746 (32.4)
Yes	2417 (59.7)	1820 (58.9)	535 (62.3)	62 (65.3)	860 (49.3)	1557 (67.6)
Social support, AVSI^d^						
High	2056 (50.8)	1676 (54.2)	338 (39.4)	42 (44.2)	915 (52.5)	1141 (49.5)
Low	1990 (49.2)	1416 (45.8)	521 (60.6)	53 (55.8)	828 (47.5)	1162 (50.5)
Social support, AVAT^d^						
High	2281 (56.4)	1657 (53.6)	570 (66.4)	54 (56.8)	917 (52.6)	1364 (59.2)
Low	1765 (43.6)	1435 (46.4)	289 (33.6)	41 (43.2)	826 (47.4)	939 (40.8)
*Outcomes*						
Hazardous alcohol use^e^						
No	2168 (53.6)	1683 (54.4)	439 (51.1)	46 (48.4)	928 (53.2)	1240 (53.8)
Yes	155 (3.8)	113 (3.7)	37 (4.3)	5 (5.3)	52 (3.0)	103 (4.5)
Missing	1723 (42.6)	1296 (41.9)	383 (44.6)	44 (46.3)	763 (43.8)	960 (41.7)
Diagnosis of alcohol use disorder^f^						
No	4014 (99.2)	3073 (99.4)	847 (98.6)	94 (98.9)	1737 (99.7)	2277 (98.9)
Yes	32 (.8)	19 (.6)	12 (1.4)	1 (1.1)	6 (.3)	26 (1.1)

^a^Defined as having high demand and low control according to Karasek–Theorell’s job–demand–control–support framework, measured in 2001–03. High/low job demand/control were dichotomized using a median or higher vs lower cut-off. Median (interquartile range—IQR) of the sum of demand and control scores were 14 (4) and 19 (4), respectively.

^b^Defined as having at least one family-related stressful life event (e.g. serious conflict with partner, death of a child, abortion) in the past 12 months, measured in 2001–03.

^c^Any diagnosis of mental (International Classification of Diseases version-10 or ICD-10 F codes) or somatic disorders (other ICD-10 codes except F, O, P, and Q codes) in inpatient healthcare during 1998–2000.

^d^Measured using Availability of Social Integration (AVSI) and Availability of Social Attachment (AVAT) scales and was dichotomized (high, low) using a median or higher vs lower cut-off value. Median (IQR) of the sum of AVSI and AVAT scores were 17 (7) and 10 (4), respectively.

^e^Alcohol Use Disorder Identification Test (AUDIT) score of ≥8 among men or ≥7 among women, measured in 2010.

^f^Any diagnosis of alcohol use disorder (ICD-10 code F10) in inpatient or specialized outpatient healthcare during 2004–14.

The crude OR between job strain and hazardous alcohol use was 1.28 (95% CI: .88–1.88) (Table 2). Adjustment for sociodemographic variables did not change the OR substantially (1.27, 95% CI: .86–1.86), but further adjustment for health-related variables and history of stress-related variables decreased it to 1.00 (95% CI: .57–1.73) (Table 2).

**Table 2:** Risk of hazardous alcohol use^a^ among individuals exposed to work-related stress (job strain^b^) and family-related stress^c^

Exposure	*n* unexposed/*n* outcomes	*n* exposed/*n* outcomes	Crude/unadjusted OR (95% CI)	Model 1^**d**^ OR (95% CI)	Model 2^**d**^ OR (95% CI)	Model 3^**e**^ OR (95% CI)
*Job strain*	1796/113	476/37	1.28 (.88–1.88)	1.27 (.86–1.86)	1.00 (.57–1.73)	0.96 (.51–1.80)
High demand	1261/82	1023/70	1.08 (.77–1.49)	1.10 (.79–1.53)	1.01 (.72–1.42)	1.00 (.70–1.43)
Low control	1145/71	1141/79	1.15 (.82–1.59)	1.09 (.77–1.53)	1.04 (.69–1.56)	1.01 (.66–1.55)
*Any family-related stress*	980/52	1343/103	1.48 (1.05–2.08)	1.49 (1.05–2.10)	1.11 (.59–2.11)	1.11 (.56–2.17)

OR, crude and multivariate-adjusted odds ratios; 95% CI, 95% confidence intervals

^a^Alcohol Use Disorder Identification Test (AUDIT) score of ≥8 among men or ≥7 among women, measured in 2010.

^b^Defined as having high demand and low control according to Karasek–Theorell’s job–demand–control–support framework, measured in 2001–03. High/low job demand/control were dichotomized using a median or higher vs lower cut-off. Median (interquartile range—IQR) of the sum of demand and control scores were 14 (4) and 19 (4), respectively.

^c^Defined as having at least one family-related stressful life event (e.g. serious conflict with partner, death of a child, abortion) in the past 12 months, measured in 2001–03.

^d^Model 2: Model 1 + additionally adjusted for health- and previous stress-related variables (stepwise): self-rated health, history of inpatient healthcare use for mental/somatic disorders, and previous job strain/family-related stress.

^e^Model 3: Model 2 + additionally adjusted for social support.

^f^Model 1: Adjusted for sociodemographic variables (stepwise): age, sex, educational level, country of birth, living with children, living with another adult, and financial difficulties in last 12 months.

The corresponding ORs in the association between family-related stressors and hazardous alcohol use were as follows: 1.48 (95% CI: 1.05–2.08) in the crude association; 1.49 (95% CI: 1.05–2.10) after adjustment for sociodemographic variables; and 1.11 (95% CI: .59–2.11) after adjustment for health-related variables and history for work- or family-related factors.

### Effect modification by sex and social support

The analyses for job strain stratified by sex and social support yielded similar results in the crude and multivariate models compared to the analysis without stratification (Table 3; unweighted results are presented as Supplementary Table S4). Regarding family-related stress, some differences between men and women in the crude point estimates were observed, although the CIs were relatively wide and overlapping; the risk of hazardous alcohol use for those with family-related stress was slightly higher among women (OR, 95% CI: 1.66, 1.02–2.71) than among men (OR, 95% CI: 1.39, .86–2.26). Among individuals with low AVAT, the risk of hazardous alcohol use was two times higher for those with family-related stress (OR, 95% CI: 2.06, 1.17–3.62) than those without family-related stress. However, these associations among women and those with low AVAT could not be detected in the fully adjusted model. No interactions with *P* < .05 between the exposures and the effect modifiers were shown (Table 4).

**Table 3:** Risk of hazardous alcohol use^a^ among the individuals exposed to job strain and family-related stress compared with the unexposed individuals, stratified by sex and social support

Exposure	*n* unexposed/*n* outcomes	*n* exposed/*n* outcomes	Crude/Unadjusted OR (95% CI)	Adjusted^**b**^ OR (95% CI)
*Job strain* *^c^*				
Sex				
Men	696/55	154/16	1.36 (.77–2.39)	1.15 (.54–2.48)
Women	1100/58	322/21	1.29 (.77–2.15)	0.88 (.42–1.85)
Social support^d^				
High AVSI	1030/58	202/15	1.44 (.80–2.57)	1.19 (.52–2.72)
Low AVSI	766/55	274/22	1.12 (.68–1.85)	0.90 (.48–1.68)
High AVAT	945/71	317/26	1.10 (.69–1.76)	0.98 (.53–1.78)
Low AVAT	851/42	159/11	1.55 (.81–2.98)	1.07 (.42–2.71)
*Any family–related stress* *^e^*				
Sex				
Men	400/28	468/44	1.39 (.86–2.26)	1.13 (.59–2.15)
Women	580/24	875/59	1.66 (1.02–2.71)	1.11 (.50–2.47)
Support^d^				
High AVSI	546/27	707/49	1.45 (.90–2.36)	1.23 (.63–2.41)
Low AVSI	434/25	636/54	1.49 (.92–2.41)	1.05 (.49–2.29)
High AVAT	489/35	801/64	1.15 (.75–1.77)	1.01 (.58–1.74)
Low AVAT	491/17	542/39	2.06 (1.17–3.62)	1.31 (.45–3.80)

OR, crude and multivariate-adjusted odds ratios; 95% CI, 95% confidence intervals.

^a^Alcohol Use Disorder Identification Test (AUDIT) score of ≥8 among men or ≥7 among women, measured in 2010.

^b^Adjusted for sociodemographic (age, sex, educational level, country of birth, living with children, living with another adult financial difficulties) and health- and previous stress-related (self-rated health, history of inpatient healthcare use for mental/somatic disorders, previous job strain/family-related stress) variables (stepwise); same variables as in Model 2 in Table 2. Sex is not adjusted for in sex-stratified analysis.

^c^Defined as having high demand and low control according to Karasek–Theorell’s job–demand–control–support framework, measured in 2001–03. High/low job demand/control were dichotomized using a median or higher vs lower cut-off. Median (interquartile range—IQR) of the sum of demand and control scores were 14 (4) and 19 (4), respectively.

^d^Measured using Availability of Social Integration (AVSI) and Availability of Social Attachment (AVAT) scales and was dichotomized (high, low) using a median or higher versus lower cut-off value. Median (IQR) of the sum of AVSI and AVAT scores were 17 (7) and 10 (4), respectively

^e^Defined as having at least one family-related stressful life event (e.g. serious conflict with partner, death of a child, abortion) in the past 12 months, measured in 2001–03.

**Table 4:** Interaction between each exposure (work-related stress/job strain^a^) and family-related stress^b^) and each effect modifier (sex and social support) for risk of hazardous alcohol use^c^

	β (standard error)	Wald chi2	*P*-value
**Interaction tests for models without adjusting for other covariates**			
Job strain*Sex	−0.075 (.398)	0.036	0.850
Job strain*Social support^d^ (AVSI)	−0.175 (.399)	0.192	0.662
Job strain*Social support^d^ (AVAT)	0.264 (.424)	0.386	0.534
Family-related stress*Sex	0.195 (.354)	0.303	0.582
Family-related stress*Social support^d^ (AVSI)	0.059 (.352)	0.028	0.867
Family-related stress*Social support^d^ (AVAT)	0.652 (.369)	3.117	0.077
**Interaction tests for models adjusting for other covariates**			
Job strain*Sex	0.234 (.305)	0.589	0.443
Job strain*Social support^d^ (AVSI)	−0.250 (.404)	0.383	0.536
Job strain*Social support^d^ (AVAT)	0.256 (.427)	0.359	0.549
Family-related stress*Sex	0.203 (.356)	0.324	0.569
Family-related stress*Social support^d^ (AVSI)	0.014 (.355)	0.002	0.969
Family-related stress*Social support^d^ (AVAT)	0.689 (.373)	3.417	0.065

^a^Defined as having high demand and low control according to Karasek–Theorell’s job–demand–control–support framework, measured in 2001–03. High/low job demand/control were dichotomized using a median or higher vs lower cut-off. Median (interquartile range—IQR) of the sum of demand and control scores were 14 (4) and 19 (4), respectively.

^b^Defined as having at least one family-related stressful life event (e.g. serious conflict with partner, death of a child, abortion) in the past 12 months, measured in 2001–03.

^c^Alcohol Use Disorder Identification Test (AUDIT) score of ≥8 among men or ≥7 among women, measured in 2010.

^d^Measured using Availability of Social Integration (AVSI) and Availability of Social Attachment (AVAT) scales and was dichotomized (high, low) using a median or higher versus lower cut-off value. Median (IQR) of the sum of AVSI and AVAT scores were 17 (7) and 10 (4), respectively.

### Secondary analyses

Overall, 32 (0.8%) individuals had a diagnosis of AUD in specialized healthcare during the follow-up. The risk (hazard ratio) of AUD was more than two times higher among those with job strain and more than three times higher for those with any family-related stress (Supplementary Table S2). This association attenuated considerably for job strain but not for family-related stressors in the multi-adjusted model (95% CI: 1.34, .57–3.15 and 2.88, 1.14–7.23, respectively).

Those reporting both job strain and family-related stress had 1.89 times (95% CI: 1.12–3.21) higher risk of hazardous alcohol use in the crude model than those reporting neither of the exposures (Supplementary Table S3). After adjustment for socioeconomic, health-related factors and history of work- and family-related stressors, the OR attenuated to 1.58 with wide CI (95% CI: .83–3.04). The results for those experiencing only work- or family-related stressors (OR, 95% CI: 1.63, 1.07–2.48 and 1.42, .66–3.07) were similar to the main analysis (Supplementary Table S3).

## Discussion

### Main findings

In this population-based longitudinal study, we investigated the relationship between work- and family-related stressors and developing hazardous alcohol use among low-risk drinkers. We found only weak evidence, with considerable uncertainty, for an association between job strain and hazardous alcohol use. In contrast, exposure to at least one family-related stressor was associated with a substantially increased risk of hazardous alcohol use. Adjusting for prior exposure to job strain and family-related stress considerably attenuated most of these associations. Stratification be sex and social support indicated that women and those with lower availability of social attachment (AVAT) had a higher risk for hazardous alcohol use when exposed to family-related stressors. However, the interaction effect was weak.

### Comparison with previous studies

Our finding of a weak and uncertain association between job strain and hazardous alcohol use is, to some extent, consistent with the inconclusive results from previous longitudinal studies (Heikkilä *et al*. 2012, Dobson *et al*. 2018, Almroth *et al*. 2022). A comprehensive meta-analysis by Heikkilä *et al*. (2012) has found a U-shaped association between job strain and alcohol use in cross-sectional studies, but the result of the longitudinal studies did not show a positive association between job strain at baseline and risk of excessive drinking during the follow-up among individuals who were nondrinkers or moderate drinkers at baseline (Heikkilä *et al*. 2012). Among the individual longitudinal studies included in the review, two studies from Finland and Belgium reported estimates similar to ours, whereas the remaining studies found either negative or no associations. Another, more recent study also observed only weak or no associations with job strain and alcohol use problems (Dobson *et al*. 2018). A register-based longitudinal study from Sweden (Almroth *et al*. 2022) including around 3 million individuals during 2005–16 reported only a weak association between high-strain jobs and a diagnosis of AUDs for men and no association for women. In our sensitivity analysis using similar register-based outcomes, we found indications that self-assessed job strain might be associated with increased healthcare use for AUD.

We found a higher risk of hazardous alcohol use for individuals exposed to at least one family-related stressful life event. Previous longitudinal studies show different results, which might be due to the substantial variation in outcome definitions and study populations across studies, which limits the possibility of direct comparisons with our findings. Studies in smaller adult community samples in the USA have reported an association between the number of general life stressors and problematic alcohol use (Cole *et al*. 1990, King *et al*. 2003). However, a study conducted among older adults (mean age 61 years) found no long-term effects of acute stressful life events on alcohol consumption patterns (Skaff *et al*. 1999). In the US-based Health and Retirement Study, widowhood was associated with increased alcohol consumption, while divorce was linked to both increased and decreased drinking (Perreira and Sloan 2001). In a cohort study of younger adults, experiencing the highest levels of psychological distress due to stressful life events was associated with alcohol abuse or dependence (AAD) (Boden *et al*. 2014). Our findings on the combined effect of experiencing both work- and family-related stress and the effect of the duration of stress are not directly comparable with other studies. These findings indicate that the type, duration, and intensity of stress might increase the allostatic load and pose a higher risk of transitioning to hazardous drinking (Keyes *et al*. 2012). Therefore, monitoring of psychological distress might be useful to identify individuals at risk.

Our study showed a slightly higher risk among women in the association between family-related stress and hazardous alcohol use. A higher risk of developing alcohol-related problems in women after stressful life events was found in other studies among employed individuals (Rospenda *et al*. 2008) and among the general population (Boden *et al*. 2014). The higher risk in women can be partly explained by the higher neurobiological sensitivity to alcohol, which can lead to the quicker development of dependence (Keyes *et al*. 2011). Contrary to these findings, in a prospective cohort study of the general Dutch population aged 45–70 years, no sex differences were observed in the association between stressors and alcohol use (Veenstra *et al*. 2007). Men reported higher alcohol use than women in response to family/interpersonal problems in an elderly US sample (Lemke *et al*. 2008), which might reflect the importance of cultural and social factors in the association. Further research is necessary to better understand the mechanisms that may explain sex differences in the relationship between family-related stressful life events and the transition to hazardous alcohol use.

We found a two-times higher risk of hazardous alcohol use among individuals with family-related stress and low qualitative social support (AVAT) than among those without family-related stress and low AVAT. We did not find similar differences concerning the quantitative aspects of social support. While our results are not directly comparable with other studies, social support and supportive resources, such as spouse, family, friends, and church, were found to be associated with stress-buffering effects and subsequent reduction of excessive drinking in response to life crisis (Jennison 1992) and negative life-events (Veenstra *et al*. 2007). Additionally, Boateng-Poku *et al*. (2020) did not find an effect of the perceived quality of instrumental and emotional social support on the association between stressful life events and alcohol use (Boateng-Poku *et al*. 2020). Our results suggest that having a supportive relationship with close relatives/friends is an important mitigating factor on the harmful effect of stressful negative life events and their impact on hazardous drinking.

### Strengths and limitations

Our study has several strengths. Due to the longitudinal study design, we could minimize bias from the bidirectional association between stress and alcohol use and avoid reverse causation. Moreover, our analyses could adjust for several key sociodemographic, health-, and previous stress-related factors. However, our findings should be interpreted in light of certain limitations. Alcohol consumption was assessed through self-reports, which, as in other population-based studies, may be subject to misclassifications due to under-reporting (Stockwell *et al*. 2004). Nevertheless, we conducted a sensitivity analysis using physician-diagnosed AUD data from healthcare registers, which confirmed the observed associations.

Although we were able to include several important stressors in our study, we could not account for intracategory variability, i.e. the lack of specific meaning within broad categories of stressful life events (Dohrenwend 2006). Individual differences in the interpretation and perception of these stressors may also exist.

Another limitation is the substantial loss to follow-up over time, which may reduce statistical power, particularly in subgroup analysis. However, by applying IPCW, we aimed to minimize bias due to potential selective loss to follow-up, based on the assumption that the covariates included in our weighting model adequately explain the probability of continued participation in subsequent study waves. Nonetheless, we cannot rule out the influence of unmeasured factors, such as motivation, stigma, or other psychosocial variables, that may also affect attrition. While these unmeasured factors are likely correlated to some extent with the covariates we adjusted for, it is unlikely that their independent effect on attrition is substantial. Still, this limitation may have led to some underestimation of the associations due to the absence of data on these unmeasured factors and potential issues with model specification when calculating the weights. The mean (standard deviation) of the stabilized weights in our IPCW model was 1.01 (0.13), which indicates good model specification (Cole and Hernán 2008). A further limitation is the higher rate of nonresponse in the PART waves. To minimize its impact on generalizability, we only included participants without a prior history of hazardous alcohol use and those employed full- or part-time at baseline. Although we adjusted for several key covariates in our analyses, residual confounding may still influence our results. For instance, we lacked data on genetic and epigenetic factors, which could affect the association between psychological distress and hazardous alcohol use. Finally, as our findings are based on participants from Stockholm, Sweden, their generalizability to other populations may be limited due to differences in work- and family-related stressors, alcohol consumption patterns, alcohol availability, and alcohol control policies.

## Conclusion

We found greater vulnerability to transitioning into hazardous alcohol use among individuals experiencing family-related stressors, with slightly pronounced effects observed among women and those with low qualitative social support. Public health efforts may be needed for the awareness of the possible consequence of family-related stress on drinking habits and these at-risk groups could benefit from intervention strategies that enhance the availability and quality of social support. Future research may investigate other risk and protective factors of developing hazardous drinking in these vulnerable populations and develop supportive tools for individuals undergoing stressful life events.

## Supplementary Material

Supplementary_data_20250811_noendnote_agaf058

## Data Availability

The data used in this study cannot be made publicly available due to privacy regulations. According to the General Data Protection Regulation, the Swedish law SFS 2018:218, the Swedish Data Protection Act, the Swedish Ethical Review Act, and the Public Access to Information and Secrecy Act, these types of sensitive data can only be made available for specific purposes, including research, that meets the criteria for access to this sort of sensitive and confidential data as determined by a legal review. Readers may contact Assistant Professor Katalin Gémes (katalin.gemes@ki.se) regarding data availability.
