# Supplementary material for: Work- and family-related stressors and risk of hazardous alcohol use: the role of social support. A cohort study in Sweden
Source: Alcohol Alcohol. 2025 Sep 16;60(6):agaf058. doi: 10.1093/alcalc/agaf058 (PMC12448858; doi:10.1093/alcalc/agaf058)
Supplement: Supplementary_data_20250811_noendnote_agaf058 [file supplementary_data_20250811_noendnote_agaf058.docx]

**Supplementary information** for the article entitled ‘Work- and family-related stressors and risk of hazardous alcohol use: the role of social support. A cohort study in Sweden’ submitted to the journal ‘Alcohol and Alcoholism’.

**Authors:** Ridwanul Amin, PhD (1, 2), Julia Spaton Goppers, MSc (1), Jette Möller, PhD (3), Karin Engström, PhD (3), Anna Sidorchuk, PhD (4, 5), Ellenor Mittendorfer–Rutz, PhD (1), Katalin Gémes, PhD (1)

**Affiliations:**

(1) Department of Clinical Neuroscience, Division of Insurance Medicine, Karolinska Institutet, Stockholm, Sweden

(2) Department of Medicine, Division of Infectious Diseases, Karolinska Institutet, Stockholm, Sweden

(3) Department of Global Public Health, Karolinska Institutet, Stockholm, Sweden

(4) Department of Clinical Neuroscience, Centre for Psychiatry Research, Karolinska Institutet

(5) Stockholm Health Care Services, Region Stockholm, Stockholm, Sweden

**Corresponding author (email address):** Ridwanul Amin ([ridwanul.amin@ki.se](mailto:ridwanul.amin@ki.se))

**Contents**

[**Supplementary methods:** 3](#_Toc200540929)

[**Supplementary Table S1:** Specific items from the PART study questionnaire that were used define job strain, family–related stress and social support. 4](#_Toc200540930)

[**Supplementary Table S2.** Covariates included in the study as potential confounders based on their known association with the exposures and the outcome and based on similar methods used in previous studies (1-8). 9](#_Toc200540931)

[**Supplementary Table S3:** Risk of hazardous alcohol use^a^ among individuals exposed to *specific job–demand–control–support framework items^b^ and specific family–related stressful life events^c^*; crude and multivariate–adjusted Odds Ratios (OR) with corresponding 95% confidence intervals (CI) 10](#_Toc200540932)

[**Supplementary Table S4:** Risk of *alcohol use disorder^a^* among individuals exposed to job strain^b^ and any family–related stress^c^; crude and multivariate–adjusted Hazard Ratios (HR) with corresponding 95% confidence intervals (CI). 12](#_Toc200540933)

[**Supplementary Table S5:** Risk of hazardous alcohol use^a^ among individuals exposed to *only job strain^b^, to only family–related stress^c^, and to both job strain and family–related stress, compared to no job strain or family-related stress*. 13](#_Toc200540934)

[**Supplementary Table S6:** Risk of hazardous alcohol use^a^ among individuals exposed to job strain^b^ and family–related stress^c^ *(results from analysis without inverse probability of censoring weighting)* 14](#_Toc200540935)

[**Supplementary Table S7.** Risk of hazardous alcohol use^a^ and alcohol use disorder^b^ among individuals exposed to job strain and family-related stress shown in crude and stepwise-multivariate-adjusted models, stratified by age and social support. 15](#_Toc200540936)

# **Supplementary methods:**

**Non-participation and attrition:** The study sample was drawn from the PART study (Swedish acronym for Mental Health, Work and Social Relations), a population-based survey conducted between 1998-2000 (PART1) among Stockholm County residents aged 20-64 years with follow-ups in 2001-2003, 2010 and 2021 (PART2-PART4, respectively). From the 19,742 randomly selected individuals who were invited to PART1, 10,441 individuals participated (Bergman et al., 2010). Non-participation was linked to lower age, male sex, lower income and educational level, non-married, country of origin outside Nordic countries, and inpatient care or disability pension due to mental disorders (Lundberg et al., 2005). Among those participated in PART1, 8631 and 5621 individuals later participated in the PART2 and PART3, respectively. Younger age, male sex, lower income and educational level, not being married, being born outside of Nordic countries, inpatient care or disability pension due to psychiatric diagnosis were associated with non-response and attrition (Lundberg et al., 2005, Bergman et al., 2010).

**Dichotomisation of job demand, control and job strain:** There is no validated cut off to guide this method. Therefore, we have followed the original recommendation of Karasek to dichotomize at the median (Karasek RA, 1990), which has been used widely used in previous research by other researchers (Kivimäki et al., 2012, Madsen et al., 2017, Almroth et al., 2022). Alternative to this method would be using it as a scale or categorizing to more than two categories. While these alternatives would have been useful to explore dose-response analysis, given the low power in our sample, these analyses would not provide meaningful results. Using other cut offs for the dichotomized variable than the one that is recommended by the developer of the questionnaire would compromise the comparability of our results, and it would not be interpretable as those cut offs would be artificial.

**Imputation methods:** Missing values for covariates and effect modifiers were imputed using multiple imputation by chained equations using ten imputed datasets (White et al., 2011), and estimates were pooled by Rubin’s rules (Rubin, 1987). Individuals with missing data on work-related stress at PART2 (n=95), and individuals with missing data on AUDIT in PART3 (n=1723) were excluded from the respective analysis.

**Inverse Probability of Censoring Weights:** We calculated the Inverse Probability of Censoring Weights (Seaman and White, 2011) to handle potential selective attrition between PART2 and PART3 including covariates that were strongly associated with dropout (age, sex, and educational level) as explanatory factors. We then stabilized the weights to minimise variance and extreme values (Seaman and White, 2011). The equation below was used to calculate the weights:

|  | conditional probability of remaining uncensored at follow-up given exposure status at baseline |
| --- | --- |
| Inverse probability of censoring weights (stabilised) = | ~~--------------------------------------------------------------~~ |
|  | conditional probability of remaining uncensored at follow-up given exposure status and covariate distribution at baseline. |

**Secondary analysis with Cox proportional hazard regression:** We defined the outcomes as diagnosis of alcohol use disorder (AUD) (ICD-10 code F10) between 1-Jan-2004 and 31-Dec-2014 in inpatient or specialized outpatient records from the National Patient Register as the outcome. The risk of AUD in the exposure groups was estimated using Cox proportional hazard regression models (one unadjusted/crude and one multivariate-adjusted model controlling for all covariates) yielding Hazard Ratios (HR and AHR, respectively) and 95% CIs. The assumption of proportional hazard was confirmed by plotting log-minus-log Kaplan–Meier survival curves. Individuals were followed from 1-Jan-2004 until visit to specialised outpatient clinics or hospitalisation for AUD, death, or end of follow-up (31-Dec-2014), whichever occurred first.

# **Supplementary Table S1:** Specific items from the PART study questionnaire that were used define job strain, family–related stress and social support.

| **Item** | **Data source (measurement timing)** | **Specific question(s) in PART** | **Response alternatives in PART** | **Definition in the current study** |
| --- | --- | --- | --- | --- |
| **Exposures** |  |  |  |  |
| Karasek-Theorell’s framework of the job-demand-control-support (Karasek RA, 1990) | PART2 (2001-2003) | Please answer your perception of the following statements about work conditions:  1. Does your workplace require you to work very fast?  2. Does your workplace require you to work very hard? 3. Does your job demand too much effort?  4. Do you have enough time to do your tasks? 5. Are there often contradicting demands in your work? 6. Are you learning new things from your job?  7. Does your job require proficiency?  8. Does your job require creativity?  9. Does your job entail repetitive tasks?  10. Do you have freedom to decide how your work is carried out?  11. Do you have freedom to decide what work to carry out? | a) yes, often  b) yes, sometimes,  c) no, seldom,  d) no, never. | High demand (yes, no) and low control, based on median or higher vs lower score of the sum of demand (Q1-Q5) and control (Q6-Q11) questions, respectively. Job strain was defined as having both high demand and low control vs not. Median (Inter-Quartile Range–IQR) of the sum of demand and control scores were 14 (4) and 19 (4), respectively. |
| Family related stress-factors (Paykel, 1978, Theorell et al., 1975) | PART2 (2001-2003) | Have you experienced any of the following events during the past 12 months?  1-3. Serious conflict with spouse/children/close relative or friend  4-6. Severe illness/accident in spouse/children/close relative or friend  7-9. Death of spouse/children/close relative or friend  10. Loss of living space  11. Abortion (own or spouse’s)  12-13. Spouse/children being subjected to serious crime (e.g. break-in or violence)  14. Children being subjected to bullying at school  15. Children having serious issues (e.g. substance abuse, repeated truancy, criminal offence).  16. Divorce/Separation | a) yes  b) no | Any family-related stress as having at least one event (yes) or not (Fandiño-Losada et al., 2013) |
| **Outcomes** |  |  |  |  |
| Hazardous alcohol use (AUDIT: primary analysis) | PART3 (2010) | 1. Have you ever drunk at least one glass of alcohol in the last 12 months (one glass refers to 33cl strong beer/45cl beer/one glass of regular wine/one small glass of strong wine/4cl of strong spirits) | a) yes  b) no | 1) no (otherwise)  2) yes (an AUDIT score of ≥8 in men and a score of ≥7 in women on a scale of 0-40) |
|  |  | 2. How often do you drink alcohol? | a) one or fewer times a month  b) two-four times a month  c) two-three times a week  d) four or more times a week |  |
|  |  | 3. How many glasses of alcohol do you drink on a typical day? | a) 1-2  b) 3-4  c) 5-6  d) 7-9  e) 10 or more |  |
|  |  | 4. How often do you drink six such "glasses" or more on the same occasion?  5. How often in the past year have you been unable to stop drinking once you started?  6. How often in the past year have you failed to do something you should have done because you were drinking?  7. How often in the past year have you needed a "drink" in the morning after a lot of drinking the day before?8. How often in the past year have you felt guilt or remorse because of your drinking?  9. How often in the past year have you drunk so much that the next day you could not remember what you had said or done? | a) never  b) more often than one times a month  c) every month  d) every week  e) daily or almost daily |  |
|  |  | 10. Have you or anyone else been injured because of your drinking?  11. Has a relative or friend, a doctor, or someone else in healthcare been worried about your drinking or suggested that you should cut down on it? | a) no  b) yes, but not in the past year  c) yes, in last year |  |
| Alcohol use disorder (sensitivity analysis) | National in- and specialised outpatient register (2004-2014) (Ludvigsson et al., 2011, Forsberg et al., 2009) | Not applicable | Not applicable |  |
| **Covariates** as potential confounders based on their known association with the exposures and the outcome and based on similar methods used in previous studies (Skaff et al., 1999, King et al., 2003, Heikkilä et al., 2012, Dobson et al., 2018, Cole et al., 1990, Boden et al., 2014, Almroth et al., 2022, Perreira and Sloan, 2001) | | | | |
| Age groups | Total Population Register | Age in years | Not applicable | Age groups in years (25-29, 30-34, 35-39, 40-44, 45-49, 50-55) |
| Sex | Total Population Register | Sex | Not applicable | 1) men  2) women |
| Educational level | PART2 (2001-2003) | Which of the following education have you attained?   - elementary - primary - junior secondary/girl - practical upper secondary (2 Years) - theoretical upper secondary (3-4 Years) - post upper secondary (2 years) - university/college | a) started b) finished | 1) compulsory school  2) upper secondary school  3) university/college; if answered ‘finished’ a certain educational level then coded as the highest finished level. If answered ‘started’ a certain level, then coded as the adjacent lower level of the highest started level. |
| Country of birth | PART2 (2001-2003) | Were you born in Sweden? | a) yes  b) no | 1) Sweden  2) Others |
| Living with another adult | PART2 (2001-2003) | Do you cohabit with an adult person? | a) no b) yes, with partner c) yes, with parents d) yes, with other adults | 1) no (response option a)  2) yes (otherwise) |
| Living with children | PART2 (2001-2003) | Are there children living in your household? | a) yes, permanently b) yes, half of the time c) yes, less than half of the time d) no, occasionally or never | 1) no (response option d)  2) yes (otherwise) |
| Financial difficulties | PART2 (2001-2003) | 1. In the past 12 months, if you suddenly faced an unforeseen situation, when you need to get 14000kr in a week, would you be able to do that? | a) yes, absolutely b) yes, probably c) no, probably not d) no | 1) no (otherwise)  2) yes if 1.c-d or 2.a-b or 3.a-b |
|  |  | 2. In the past 12 months, were you forced to borrow money from relatives/friends to bear cost of food or housing? | a) yes, on several occasions b) yes, on one occasion c) no |  |
|  |  | 3. In the past 12 months, were you forced to get help from social welfare to bear cost of food or housing? | a) yes, on several occasions b) yes, on one occasion c) no |  |
| Self-reported health | PART2 (2001-2003) | How do you rate your current health status? | a) very good b) good c) average d) bad e) very bad | 1) good-average (response options a-c) 2) bad-very bad (otherwise) |
| Previous healthcare use for mental disorders | National inpatient register (1998-2000) | Not applicable | Not applicable | Any hospitalisation with any ICD-10 F codes as yes and no, otherwise. |
| Previous healthcare use for somatic disorders | National inpatient register (1998-2000) | Not applicable | Not applicable | Any hospitalisation with any ICD-10 except codes for mental disorders (F), pregnancy, childbirth and the puerperium (O), certain conditions originating in the perinatal period (P), and congenital malformations, deformations and chromosomal abnormalities (Q) as yes and no, otherwise. |
| Previous job strain | PART1 (1998-2000) | Same as exposure measured in PART2 | Same as exposure measured in PART2 | Same as exposure measured in PART2 |
| Previous family-related stress | PART1 (1998-2000) | Same as exposure measured in PART2 | Same as exposure measured in PART2 | Same as exposure measured in PART2 |
| **Effect modifier** |  |  |  |  |
| AVSI (Availability of Social Integration) – quantitative aspects of social support | PART2 (2001-2003) | D1: How many people with the same interests as you do you know and are in contact with?  D2: How many people who you know, do you see or speak to during a regular week?  D3: How many friends do you have who can come home to you anytime and feel at home?  D4: How many are there among your friends with whom you can speak openly without thinking about what you say? | a) no one,  b) 1-2  c) 3-5  d) 6-10  e) 11-15  f) more than 15 | Social support - low vs high AVSI social support based on median or higher vs lower score of the sum of all AVSI questions. Median (IQR) of the sum of AVSI scores was 17 (7). |
| AVAT (Availability of Social Attachment) - qualitative aspects of social support | PART2 (2001-2003) | D5: There is a special person who I feel I can really get support from.  D6: There is a special person who feel they are very close to me.  D7: Other people show appreciation for what I do.  D8: There are people in my surrounding who I can easily ask for help.  D9: Besides for those at home, are there others who I can turn to if I am in trouble, someone I can easily meet who I trust and who can really help me during difficult times. | a) very accurate,  b) rather accurate  c) not very accurate  d) not accurate at all | Social support - low vs high AVAT social support based on median or higher vs lower score of the sum of all AVAT questions. Median (IQR) of the sum of AVAT scores was 10 (4). |
|  | PART2 (2001-2003) | D10: Are you rooted and feel strong connection to the neighbourhood in which you live?  D11: Do you feel strong connection to your family and relatives? | a) to a large extent,  b) to some extent,  c) not particularly,  d) not at all |  |

# **Supplementary Table S2:** Risk of *alcohol use disorder^a^* among individuals exposed to job strain^b^ and any family–related stress^c^; crude and multivariate–adjusted Hazard Ratios (HR) with corresponding 95% confidence intervals (CI).

| Exposure | Number of outcomes (rate/100000 person–years) among unexposed | Number of outcomes (rate/100000 person–years) among exposed | Crude/Unadjusted  HR (95% CI) | Adjusted^d^  HR (95% CI) |
| --- | --- | --- | --- | --- |
| *Job strain* | 19 (56.4) | 12 (128.6) | 2.28 (1.11–4.70) | 1.34 (0.57–3.15) |
| *Any family–related stress* | 6 (31.6) | 26 (103.8) | 3.29 (1.35–7.99) | 2.88 (1.14–7.23) |

^a^ Any primary or secondary diagnosis of alcohol use disorder (International Classification of Diseases version–10 or ICD–10 code F10) between 1–Jan–2004 and 31–Dec–2014 in inpatient or specialized outpatient settings

^b^ Defined as having high demand and low control according to Karasek–Theorell’s job–demand–control–support framework, measured in 2001–2003. High/low job demand/control were dichotomised using a median or higher vs lower cut-off. Median (Inter-Quartile Range-IQR) of the sum of demand and control scores were 14 (4) and 19 (4), respectively.

^c^ Defined as having at least one family–related stressful life event (e.g. serious conflict with partner, death of a child, abortion etc.) in the past 12 months, measured in 2001–2003

^d^ Adjusted for sociodemographic (age, sex, educational level, country of birth, living with children, living with another adult, financial difficulties) and health– and previous stress–related (self–rated health, history of inpatient healthcare use for mental/somatic disorders, previous job strain/family–related stress) variables (stepwise)

# **Supplementary Table S3:** Risk of hazardous alcohol use^a^ among individuals exposed to *only job strain^b^, to only family–related stress^c^, and to both job strain and family–related stress, compared to no job strain or family-related stress*.

| **Exposure** | **Individuals (n)** | **Outcomes (n)** | **Crude/unadjusted** **OR (95% CI)** | **Model 1^d^**  **OR (95% CI)** | **Model 2^e^**  **OR (95% CI)** | **Model 3^f^**  **OR (95% CI)** |
| --- | --- | --- | --- | --- | --- | --- |
| *No job strain or family–related stress* | 1350 | 37 | 1 | 1 | 1 | 1 |
| *Only job strain* | 355 | 13 | 1.44 (0.76–2.72) | 1.57 (0.81–3.02) | 1.42 (0.66–3.07) | 1.21 (0.50–2.90) |
| *Only family–related stress* | 1742 | 76 | 1.57 (1.05–2.36) | 1.64 (1.08–2.47) | 1.63 (1.07–2.48) | 1.56 (1.01–2.42) |
| *Both job strain and family–related stress* | 504 | 24 | 1.89 (1.12–3.21) | 1.91 (1.10–3.31) | 1.58 (0.83–3.04) | 1.47 (0.68–3.19) |

^a^ Alcohol Use Disorder Identification Test (AUDIT) score of ≥8 among men or ≥7 among women, measured in 2010
^b^ Defined as having high demand and low control according to Karasek–Theorell’s job–demand–control–support framework, measured in 2001–2003. High/low job demand/control were dichotomised using a median or higher vs lower cut-off. Median (Inter-Quartile Range-IQR) of the sum of demand and control scores were 14 (4) and 19 (4), respectively.
^c^ Defined as having at least one family–related stressful life event (e.g. serious conflict with partner, death of a child, abortion etc.) in the past 12 months, measured in 2001–2003
^d^ Model 1: Adjusted for sociodemographic variables (stepwise): age, sex, educational level, country of birth, living with children, living with another adult, financial difficulties in last 12 months
^e^ Model 2: Model 1 + additionally adjusted for health– and previous stress–related variables (stepwise): self–rated health, history of inpatient healthcare use for mental/somatic disorders, previous job strain/family–related stress
^f^ Model 3: Model 2 + additionally adjusted for social support
OR – crude and multivariate–adjusted Odds Ratios; 95% CI – 95% Confidence Intervals

# **Supplementary Table S4:** Risk of hazardous alcohol use^a^ among individuals exposed to job strain^b^ and family–related stress^c^ *(results from analysis without inverse probability of censoring weighting)*

| **Exposure** | **n unexposed /n outcomes** | **n exposed** **/n outcomes** | **Crude/unadjusted**  **OR (95% CI)** | **Model 1^d^**  **OR (95% CI)** | **Model 2^e^**  **OR (95% CI)** | **Model 3^f^**  **OR (95% CI)** |  |
| --- | --- | --- | --- | --- | --- | --- | --- |
| *Job strain* | 1796/113 | 476/37 | 1.26 (0.85–1.85) | 1.24 (0.84–1.84) | 0.95 (0.54–1.67) | 0.92 (0.49–1.73) |  |
| High demand | 1261/82 | 1023/70 | 1.06 (0.76–1.47) | 1.08 (0.77–1.51) | 0.99 (0.70–1.40) | 0.98 (0.69–1.40) |  |
| Low control | 1145/71 | 1141/79 | 1.13 (0.81–1.57) | 1.07 (0.76–1.51) | 1.00 (0.66–1.50) | 0.97 (0.63–1.50) |  |
| *Any family–related stress* | 980/52 | 1343/103 | 1.48 (1.05–2.09) | 1.50 (1.06–2.13) | 1.11 (0.57–2.15) | 1.10 (0.55–2.22) |  |

^a^ Alcohol Use Disorder Identification Test (AUDIT) score of ≥8 among men or ≥7 among women, measured in 2010

^b^ Defined as having high demand and low control according to Karasek–Theorell’s job–demand–control–support framework, measured in 2001–2003. High/low job demand/control were dichotomised using a median or higher vs lower cut-off. Median (Inter-Quartile Range-IQR) of the sum of demand and control scores were 14 (4) and 19 (4), respectively

^c^ Defined as having at least one family–related stressful life event (e.g. serious conflict with partner, death of a child, abortion etc.) in the past 12 months, measured in 2001–2003

^d^ Model 1: Adjusted for sociodemographic variables (stepwise): age, sex, educational level, country of birth, living with children, living with another adult, financial difficulties in last 12 months

^e^ Model 2: Model 1 + additionally adjusted for health– and previous stress–related variables (stepwise): self–rated health, history of inpatient healthcare use for mental/somatic disorders, previous job strain/family–related stress

^f^ Model 3: Model 2 + additionally adjusted for social support

OR – crude and multivariate–adjusted Odds Ratios; 95% CI – 95% Confidence Intervals

# **Supplementary Table S5.** Risk of hazardous alcohol use^a^ and alcohol use disorder^b^ among individuals exposed to job strain and family-related stress shown in crude and stepwise-multivariate-adjusted models, stratified by age and social support.

| Job strain^c^ |  |  |  |  |  |  |  |  |
| --- | --- | --- | --- | --- | --- | --- | --- | --- |
| Outcome | AUDIT | | | | | | | AUD |
| Stratification | None | Sex | | Social support (AVSI)^c^ | | Social support (AVAT) ^c^ | |  |
|  |  | Men | Women | High | Low | High | Low |  |
| Models |  |  |  |  |  |  |  |  |
| Crude/unadjusted | 1.28 (0.88–1.88) | 1.36 (0.77–2.39) | 1.29 (0.77–2.15) | 1.44 (0.80–2.57) | 1.12 (0.68–1.85) | 1.10 (0.69–1.76) | 1.55 (0.81–2.98) | 2.28 (1.11–4.70) |
| +age | 1.30 (0.89–1.91) | 1.36 (0.77–2.42) | 1.31 (0.78–2.19) | 1.48 (0.83–2.66) | 1.13 (0.68–1.87) | 1.14 (0.71–1.82) | 1.51 (0.78–2.91) | 2.30 (1.12–4.72) |
| +sex | 1.33 (0.91–1.95) | NA | NA | 1.50 (0.84–2.70) | 1.16 (0.70–1.93) | 1.17 (0.73–1.87) | 1.56 (0.81–3.03) | 2.33 (1.13–4.83) |
| +educational level | 1.31 (0.89–1.91) | 1.30 (0.73–2.32) | 1.29 (0.77–2.17) | 1.43 (0.79–2.57) | 1.17 (0.70–1.94) | 1.14 (0.71–1.84) | 1.52 (0.78–2.95) | 2.33 (1.13–4.83) |
| +country of birth | 1.31 (0.89–1.92) | 1.31 (0.73–2.34) | 1.29 (0.77–2.17) | 1.42 (0.79–2.55) | 1.14 (0.68–1.91) | 1.15 (0.72–1.85) | 1.50 (0.77–2.93) | 2.29 (1.10–4.75) |
| +living with another adult | 1.31 (0.89–1.92) | 1.33 (0.74–2.38) | 1.30 (0.77–2.18) | 1.41 (0.78–2.54) | 1.14 (0.68–1.91) | 1.15 (0.72–1.85) | 1.51 (0.78–2.95) | 2.14 (1.03–4.45) |
| +living with children | 1.31 (0.89–1.92) | 1.33 (0.74–2.40) | 1.31 (0.78–2.20) | 1.40 (0.78–2.53) | 1.13 (0.68–1.89) | 1.16 (0.72–1.86) | 1.56 (0.80–3.06) | 2.16 (1.04–4.49) |
| +financial difficulties | 1.27 (0.86–1.86) | 1.33 (0.74–2.39) | 1.23 (0.73–2.08) | 1.41 (0.78–2.54) | 1.10 (0.66–1.85) | 1.15 (0.71–1.84) | 1.42 (0.72–2.81) | 2.08 (0.99–4.34) |
| +self–rated health | 1.24 (0.84–1.83) | 1.31 (0.73–2.36) | 1.21 (0.72–2.05) | 1.42 (0.79–2.57) | 1.03 (0.61–1.74) | 1.13 (0.70–1.82) | 1.40 (0.71–2.78) | 1.89 (0.91–3.95) |
| +history of inpatient healthcare use for mental disorders | 1.24 (0.84–1.83) | 1.31 (0.73–2.36) | 1.21 (0.71–2.05) | 1.42 (0.79–2.57) | 1.03 (0.61–1.74) | 1.13 (0.70–1.82) | 1.40 (0.70–2.78) | 1.89 (0.90–3.95) |
| +history of inpatient healthcare use for somatic disorders | 1.24 (0.84–1.83) | 1.31 (0.73–2.36) | 1.20 (0.71–2.03) | 1.40 (0.78–2.54) | 1.02 (0.61–1.73) | 1.13 (0.70–1.82) | 1.38 (0.69–2.75) | 1.99 (0.95–4.17) |
| +previous job strain | 0.99 (0.57–1.74) | 1.16 (0.54–2.50) | 0.87 (0.41–1.83) | 1.19 (0.52–2.72) | 0.90 (0.48–1.68) | 0.97 (0.53–1.77) | 1.07 (0.42–2.74) | 1.32 (0.56–3.12) |
| +previous family–related stress | 1.00 (0.57–1.73) | 1.15 (0.54–2.48) | 0.88 (0.42–1.85) | 1.19 (0.52–2.72) | 0.90 (0.48–1.68) | 0.98 (0.53–1.78) | 1.07 (0.42–2.71) | 1.34 (0.57–3.15) |
| Family–related stress^d^ |  |  |  |  |  |  |  |  |
| Crude/unadjusted | 1.48 (1.05–2.08) | 1.39 (0.86–2.26) | 1.66 (1.02–2.71) | 1.45 (0.90–2.36) | 1.49 (0.92–2.41) | 1.15 (0.75–1.77) | 2.06 (1.17–3.62) | 3.29 (1.35–7.99) |
| +age | 1.48 (1.05–2.08) | 1.40 (0.86–2.29) | 1.64 (1.01–2.68) | 1.46 (0.90–2.37) | 1.49 (0.92–2.41) | 1.16 (0.75–1.78) | 2.06 (1.17–3.64) | 3.23 (1.32–8.01) |
| +sex | 1.52 (1.08–2.14) | NA | NA | 1.46 (0.90–2.38) | 1.60 (0.98–2.60) | 1.21 (0.78–1.87) | 2.12 (1.20–3.74) | 3.30 (1.36–8.03) |
| +educational level | 1.53 (1.08–2.16) | 1.42 (0.87–2.32) | 1.65 (1.01–2.70) | 1.49 (0.91–2.43) | 1.58 (0.97–2.58) | 1.21 (0.78–1.87) | 2.10 (1.19–3.72) | 3.30 (1.36–8.03) |
| +country of birth | 1.53 (1.09–2.16) | 1.44 (0.88–2.34) | 1.66 (1.01–2.70) | 1.51 (0.93–2.47) | 1.57 (0.97–2.57) | 1.22 (0.79–1.89) | 2.10 (1.19–3.71) | 3.28 (1.35–7.98) |
| +living with another adult | 1.53 (1.09–2.16) | 1.44 (0.88–2.34) | 1.65 (1.01–2.69) | 1.51 (0.93–2.47) | 1.57 (0.96–2.56) | 1.22 (0.79–1.89) | 2.08 (1.17–3.68) | 3.18 (1.30–7.75) |
| +living with children | 1.53 (1.08–2.16) | 1.46 (0.90–2.39) | 1.61 (0.98–2.64) | 1.52 (0.93–2.48) | 1.54 (0.94–2.51) | 1.24 (0.80–1.92) | 2.04 (1.15–3.62) | 3.18 (1.30–7.78) |
| +financial difficulties | 1.49 (1.05–2.10) | 1.45 (0.88–2.36) | 1.55 (0.95–2.55) | 1.47 (0.90–2.40) | 1.51 (0.93–2.48) | 1.22 (0.78–1.89) | 1.99 (1.12–3.54) | 3.11 (1.27–7.60) |
| +self–rated health | 1.47 (1.04–2.08) | 1.43 (0.88–2.35) | 1.54 (0.94–2.53) | 1.48 (0.90–2.42) | 1.46 (0.89–2.40) | 1.20 (0.77–1.87) | 1.98 (1.11–3.53) | 2.81 (1.15–6.88) |
| +history of inpatient healthcare use for mental disorders | 1.48 (1.05–2.09) | 1.44 (0.88–2.35) | 1.55 (0.95–2.54) | 1.48 (0.90–2.42) | 1.47 (0.89–2.40) | 1.20 (0.78–1.87) | 1.99 (1.12–3.54) | 2.82 (1.15–6.90) |
| +history of inpatient healthcare use for somatic disorders | 1.48 (1.04–2.09) | 1.44 (0.88–2.35) | 1.55 (0.94–2.54) | 1.47 (0.90–2.41) | 1.49 (0.91–2.44) | 1.21 (0.78–1.88) | 1.98 (1.11–3.53) | 2.77 (1.13–6.80) |
| +previous job strain | 1.48 (1.05–2.09) | 1.39 (0.85–2.29) | 1.54 (0.94–2.53) | 1.53 (0.93–2.51) | 1.46 (0.89–2.40) | 1.20 (0.77–1.86) | 2.07 (1.16–3.70) | 2.79 (1.11–7.00) |
| +previous family–related stress | 1.11 (0.59–2.11) | 1.13 (0.59–2.15) | 1.11 (0.50–2.47) | 1.23 (0.63–2.41) | 1.05 (0.49–2.29) | 1.01 (0.58–1.74) | 1.31 (0.45–3.80) | 2.79 (1.11–7.00) |

^a^ Defined as Alcohol Use Disorder Identification Test (AUDIT) score of ≥8 among men or ≥7 among women, measured in 2010; ‘no job strain’ is the reference category for each column
^b^ Any primary or secondary diagnosis of alcohol use disorder (International Classification of Diseases version-10 or ICD-10 code F10) between 1-Jan-2004 and 31-Dec-2014 in inpatient or specialized outpatient settings
^c^ Defined as having high demand and low control according to Karasek-Theorell’s job-demand-control-support framework, measured in 2001-2003. High/low job demand/control were dichotomised using a median or higher vs lower cut-off. Median (Inter-Quartile Range-IQR) of the sum of demand and control scores were 14 (4) and 19 (4), respectively.
^d^ Defined as having at least one family-related stressful life event (e.g. serious conflict with partner, death of a child, abortion etc.) in the past 12 months, measured in 2001-2003; ‘no family-related stress’ is the reference category for each column
^e^ Measured using Availability of Social Integration (AVSI) and Availability of Social Attachment (AVAT) scales and was dichotomised (high, low) using a median or higher versus lower cut-off value. Median (IQR) of the sum of AVSI and AVAT scores were 17 (7) and 10 (4), respectively
OR - crude and multivariate-adjusted Odds Ratios; HR - crude and multivariate-adjusted Hazard Ratios; 95% CI – 95% Confidence Intervals; NA – Not Applicable when stratifying for sex

**References**

SVENSK NATIONELL DATATJÄNST*. Psykisk hälsa - Arbete - Relationer (PART) - Enkätundersökningarna 1-3.* [*https://snd.se/sv/catalogue/dataset/snd0784-1*](https://snd.se/sv/catalogue/dataset/snd0784-1) *(26 September 2024, date last accessed)*

ALMROTH, M., HEMMINGSSON, T., SÖRBERG WALLIN, A., KJELLBERG, K. & FALKSTEDT, D. 2022. Psychosocial workplace factors and alcohol-related morbidity: a prospective study of 3 million Swedish workers. *Eur J Public Health,* 32**,** 366-371.

BERGMAN, P., AHLBERG, G., FORSELL, Y. & LUNDBERG, I. 2010. Non-Participation in the Second Wave of the Part Study on Mental Disorder and Its Effects on Risk Estimates. *Int J Soc Psychiatr,* 56**,** 119-132.

BODEN, J. M., FERGUSSON, D. M. & HORWOOD, L. J. 2014. Associations between exposure to stressful life events and alcohol use disorder in a longitudinal birth cohort studied to age 30. *Drug Alcohol Depend,* 142**,** 154-160.

COLE, G., TUCKER, L. & FRIEDMAN, G. M. 1990. Relationships among measures of alcohol drinking behavior, life-events and perceived stress. *Psychol Rep,* 67**,** 587-91.

DOBSON, K. G., IBRAHIM, S., GILBERT-OUIMET, M., MUSTARD, C. A. & SMITH, P. M. 2018. Association between psychosocial work conditions and latent alcohol consumption trajectories among men and women over a 16-year period in a national Canadian sample. *J Epidemiol Community Health,* 72**,** 113-120.

FANDIÑO-LOSADA, A., FORSELL, Y. & LUNDBERG, I. 2013. Demands, skill discretion, decision authority and social climate at work as determinants of major depression in a 3-year follow-up study. *Int Arch Occup Environ Health,* 86**,** 591-605.

SOCIALSTYRELSEN. *Kvalitet och innehåll i patientregistret. Utskrivningar från slutenvården 1964–2007 och besök i specialiserad öppenvård (exklusive primärvårdsbesök) 1997–2007.* <https://www.socialstyrelsen.se/publikationer/kvalitet-och-innehall-i-patientregistret--utskrivningar-fran-slutenvarden-1964-2007-och-besok-i-specialiserad-oppenvard-exklusive-primarvardsbesok-1997-2007-2009-125-15/> (26 September 2024, date last accessed)

HEIKKILÄ, K., NYBERG, S. T., FRANSSON, E. I. et al. Job Strain and Alcohol Intake: A Collaborative Meta-Analysis of Individual-Participant Data from 140 000 Men and Women. *PLOS ONE,* 7**,** e40101.

KARASEK R. 1990. *Healthy work: Stress, productivity and the reconstruction of working life*, New York: NY Basic Books.

KING, A. C., BERNARDY, N. C. & HAUNER, K. 2003. Stressful events, personality, and mood disturbance: gender differences in alcoholics and problem drinkers. *Addict Behav,* 28**,** 171-87.

KIVIMÄKI, M., NYBERG, S. T., BATTY, G. D. et al. 2012. Job strain as a risk factor for coronary heart disease: a collaborative meta-analysis of individual participant data. *Lancet,* 380**,** 1491-7.

LUDVIGSSON, J. F., ANDERSSON, E., EKBOM, A. et al. 2011. External review and validation of the Swedish national inpatient register. *BMC Public Health,* 11**,** 450.

LUNDBERG, I., DAMSTRÖM THAKKER, K., HÄLLSTRÖM, T. & FORSELL, Y. 2005. Determinants of non-participation, and the effects of non-participation on potential cause-effect relationships, in the PART study on mental disorders. *Soc Psychiatry Psychiatr Epidemiol,* 40**,** 475-83.

MADSEN, I. E. H., NYBERG, S. T., MAGNUSSON HANSON, L. L. et al. 2017. Job strain as a risk factor for clinical depression: systematic review and meta-analysis with additional individual participant data. *Psychol Med,* 47**,** 1342-1356.

PAYKEL, E. S. 1978. Contribution of life events to causation of psychiatric illness. *Psychol Med,* 8**,** 245-53.

PERREIRA, K. M. & SLOAN, F. A. 2001. Life events and alcohol consumption among mature adults: a longitudinal analysis. *J Stud Alcohol,* 62**,** 501-508.

RUBIN, D. B. 1987. Applied probability and statistics. In: Multiple Imputation for Nonresponse in Surveys. Wiley series in probability and mathematical statistics. JOHN WILEY & SONS, New York, Chichester, Brisbane, Toronto, Singapore.

SEAMAN, S. R. & WHITE, I. R. 2011. Review of inverse probability weighting for dealing with missing data. *Stat Methods Med Res,* 22**,** 278-295.

SKAFF, M. M., FINNEY, J. W. & MOOS, R. H. 1999. Gender differences in problem drinking and depression: different "vulnerabilities?". *Am J Community Psychol,* 27**,** 25-54.

THEORELL, T., LIND, E. & FLODÉRUS, B. 1975. The relationship of disturbing life-changes and emotions to the early development of myocardial infarction and other serious illnesses. *Int J Epidemiol,* 4**,** 281-93.

WHITE, I. R., ROYSTON, P. & WOOD, A. M. 2011. Multiple imputation using chained equations: Issues and guidance for practice. *Stat Med,* 30**,** 377-399.
